# Recruitment Strategies and Lessons Learned from the Children's Healthy Living Program Prevalence Survey

**DOI:** 10.3934/publichealth.2016.1.140

**Published:** 2016-03-21

**Authors:** Marie K. Fialkowski, Ashley Yamanaka, Lynne R. Wilkens, Kathryn L. Braun, Jean Butel, Reynolette Ettienne, Katalina McGlone, Shelley Remengesau, Julianne M. Power, Emihner Johnson, Daisy Gilmatam, Travis Fleming, Mark Acosta, Tayna Belyeu-Camacho, Moria Shomour, Cecilia Sigrah, Claudio Nigg, Rachel Novotny

**Affiliations:** 1 Department of Human Nutrition, Food, and Animal Sciences, University of Hawai‘i at Mānoa, Honolulu, HI; 2 Epidemiology Program, University of Hawai‘i Cancer Center, Honolulu HI; 3 Office of Public Health Studies, University of Hawai‘i at Mānoa, Honolulu, HI; 4 Center for Alaska Native Health Research, University of Alaska Fairbanks, AK; 5 Island Food Community of Pohnpei, Kolonia, Pohnpei, Federated States of Micronesia; 6 Yap State Hospital, Colonia, Yap, Federated States of Micronesia; 7 Community and Natural Resources Division, American Samoa Community College, Mesepa, AS; 8 College of Natural and Applied Sciences, University of Guam, Mangilao, GU; 9 Cooperative Research, Extension, and Education Service, Northern Marianas College, Saipan, MP; 10 Chuuk State Department of Health Services, Weno, Chuuk, Federated States of Micronesia; 11 Kosrae State Hospital, Tofol, Kosrae, Federated States of Micronesia

**Keywords:** Prevalence, multi-site, recruitment, Pacific, childhood, obesity

## Abstract

The US Affiliated Pacific region's childhood obesity prevalence has reached epidemic proportions. To guide program and policy development, a multi-site study was initiated, in collaboration with partners from across the region, to gather comprehensive information on the regional childhood obesity prevalence. The environmental and cultural diversity of the region presented challenges to recruiting for and implementing a shared community-based, public health research program. This paper presents the strategies used to recruit families with young children (n = 5775 for children 2 – 8 years old) for obesity-related measurement across eleven jurisdictions in the US Affiliated Pacific Region. Data were generated by site teams that provided summaries of their recruitment strategies and lessons learned. Conducting this large multi-site prevalence study required considerable coordination, time and flexibility. In every location, local staff knowledgeable of the community was hired to lead recruitment, and participant compensation reflected jurisdictional appropriateness (e.g., gift cards, vouchers, or cash). Although recruitment approaches were site-specific, they were predominantly school-based or a combination of school- and community-based. Lessons learned included the importance of organization buy-in; communication, and advance planning; local travel and site peculiarities; and flexibility. Future monitoring of childhood obesity prevalence in the region should consider ways to integrate measurement activities into existing organizational infrastructures for sustainability and cost-effectiveness, while meeting programmatic (e.g. study) goals.

## 1. Introduction

The prevalence of adult and child obesity has risen to epidemic proportions [1] and is particularly high in Pacific Islander populations [2],[3]. These trends in Pacific Islanders, especially those of the US Affiliated Pacific, have led to a declaration of a state of emergency for the region [4]. If not prevented in childhood, obesity often tracks into adulthood [5] and increases risk for poor health in adulthood [6]–[9].

Jurisdiction-level efforts have been implemented in the US Affiliated Pacific to address rising levels of obesity [10]–[19], but are difficult to sustain due to limited resources in the region [4]. A recent meta-analysis on the prevalence of child obesity in the region suggests that rates increase at elementary school entry [20], emphasizing a need to evaluate and address the issue in this pivotal age group. However, even when programs are in place, systematic evaluation of child-obesity-reduction efforts across the US Affiliated Pacific has been hampered by the lack of local nutrition monitoring and surveillance systems [21],[22].

The US Affiliated Pacific region is spread across the vast Pacific Ocean covering a greater distance than does the contiguous US on land (See Table 1). This region includes the US states of Hawai‘i and Alaska, the US territories of American Samoa and Guam, the US Commonwealth of the Northern Mariana Islands (CNMI), and the countries of the Freely Associated States of Micronesia (FAS) (Federated States of Micronesia [FSM: Pohnpei, Chuuk, Yap, Kosrae], Republic of the Marshall Islands [RMI], and the Republic of Palau) that are in a Compact of Free Association with the US government [23]. Each of these jurisdictions is ethnically, culturally, and environmentally distinct. For example, the indigenous populations of American Samoa and Hawai‘i are of Polynesian descent, while those of Guam and the FAS are of Micronesian descent. The jurisdictions range considerably in topography, from low-lying atolls (RMI), to high volcanic islands (Hawai‘i) to temperate rainforests (Alaska). However, the population size in this region is small, from the largest of 1.4 million in Hawai‘i [24] to 21,000 in Palau [25]. The population size, combined with the vast area, makes systematic comprehensive public health monitoring for program and policy development difficult in this region.

In response to a call related to childhood obesity prevention from the US Department of Agriculture in 2011, stakeholders at institutions from across the US Affiliated Pacific region joined together to establish the Children's Healthy Living (CHL) Program for remote underserved populations of the Pacific region [26]. The mission of CHL is to elevate the capacity of the Pacific region, to build and sustain a healthy food and physical environment to help maintain healthy weight and prevent obesity among young children. One important component of the CHL activities was to conduct a prevalence survey on childhood obesity across all eleven jurisdictions (Alaska, American Samoa, CNMI, Chuuk, Guam, Hawai‘i, Kosrae, Pohnpei, RMI, Republic of Palau, and Yap) in the region [27]. Results from this prevalence survey can be used to develop future health promotion programs and motivate policy changes in the region.

Embarking on a prevalence survey required a coordinated effort among partners across the region. This was particularly important given the goal to recruit over 5,000 children between the ages of 2–8 years for—obesity-related measurement [27]. There is limited literature available on the recruitment processes needed for community-based studies [28],[29] within this target population (young children in the Pacific region) [30]. Although there are publications related to recruiting older adults [31]–[35], underrepresented minority adults [36],[37] and clinical trial participants [38], there is nothing available about recruiting in the Pacific region. Providing information on the recruitment processes used in a region as diverse as the Pacific is important, as it has been found that engaging ethnic minorities in research may be difficult due to previous mistreatment and distrust [39]–[41]. Also, some strategies used in developed countries (e.g., media, mailings, and record searches) [28] would likely not work in low-resource, rural communities of the Pacific.

Therefore, the purpose of this paper is to document the recruitment processes, strategies, and lessons learned from conducting a multi-jurisdiction childhood obesity prevalence survey among children across the US Affiliated Pacific region. This paper will serve as a reference for others seeking to conduct public health research in resource-limited and geographically isolated locations.

**Table 1.** Geographic Location and Description of Jurisdictions in the US Affiliated Pacific Region Participating in the Children's Healthy Living Program Childhood Obesity Prevalence Survey.

Geographic Location	Jurisdiction Description [42]
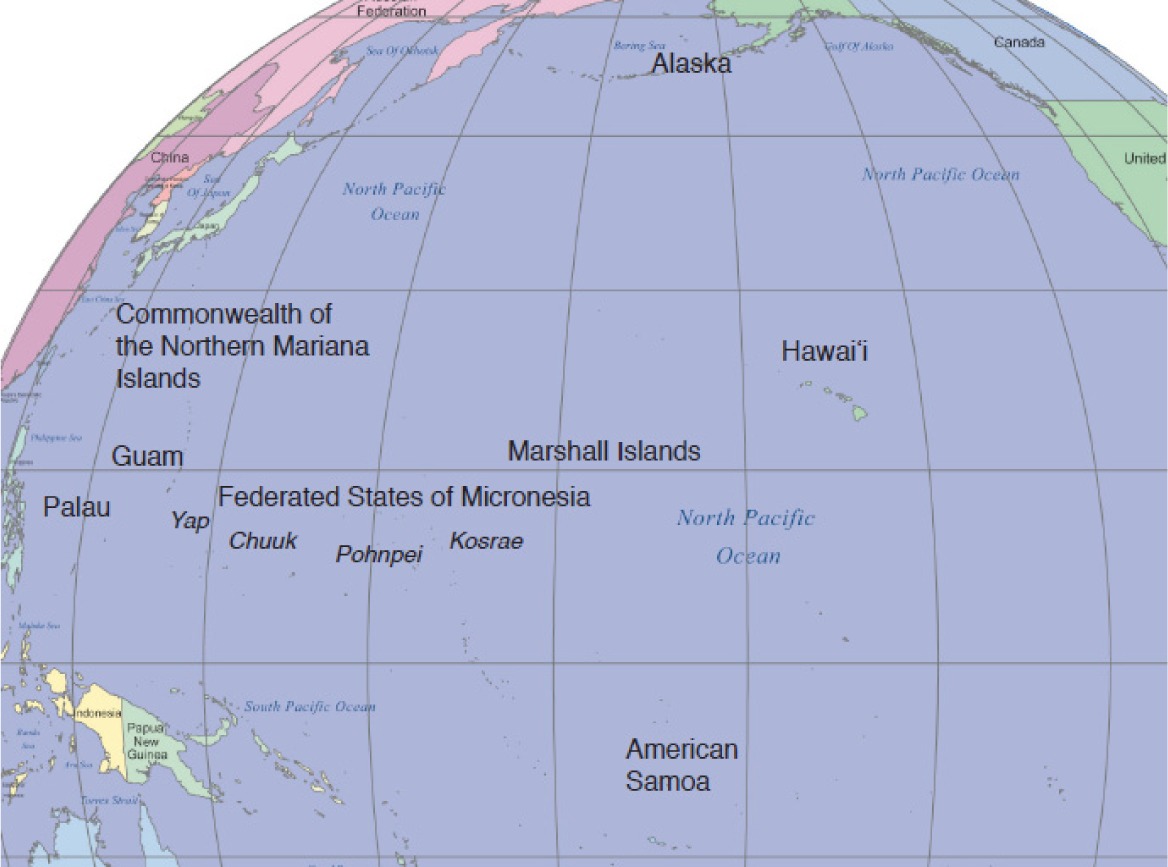	**Alaska.** US state located in the northwest of the North American continent in the Northwest Pacific Ocean.
	**American Samoa.** Territory of the US consisting of five main islands and two coral atolls located in the South Pacific Ocean.
	**Commonwealth of the Northern Mariana Islands.** US commonwealth territory of 15 volcanic islands in the Western Pacific Ocean. Consists of three major inhabited islands Saipan, Tinian, and Rota.
	**Chuuk.** State in the Federated States of Micronesia in the Western Pacific Ocean, consisting of seven main islands. In a Compact of Free Association with the US.
	**Guam.** Territory of the US in the Western Pacific Ocean.
	**Hawai‘i.** US state consisting of eight main islands in the Central Pacific Ocean.
	**Republic of Palau.** Island country in the Western Pacific Ocean consisting of 500 islands. In a Compact of Free Association with the US.
	**Kosrae.** Easternmost state of the Federated States of Micronesia in the Western Pacific Ocean. In a Compact of Free Association with the US.
	**Pohnpei.** State in the Federated States of Micronesia in the Western Pacific Ocean. In a Compact of Free Association with the US.
	**Republic of the Marshall Islands.** Island country consisting of 29 coral atolls and 1,156 individual islands and islets in the Western Pacific Ocean. In a Compact of Free Association with the US.
	**Yap.** Westernmost state of the Federated States of Micronesia in the Western Pacific Ocean. In a Compact of Free Association with the US.

## 2. Materials and Methods

### 2.1. Study Design

The prevalence study was conducted in the following eleven jurisdictions of the US Affiliated Pacific region: Alaska, American Samoa, CNMI, FSM (Chuuk, Kosrae, Pohnpei, Yap), Guam, Hawai‘i, RMI, and Palau. In Alaska, American Samoa, CNMI, Guam, and Hawai‘i, the prevalence survey was implemented as a part of the baseline measures for a community randomized intervention trial [27]. A prevalence survey only was conducted in each of the four states of the FSM and in the RMI and Palau.

The multi-jurisdiction prevalence study (NIH clinical trial # NCT01881373 [clinicaltrials.gov]) was conducted between Fall 2012 and Spring 2015, with a recruitment goal of 5,000+ children [27]. Sample size requirements were considered separately for the five intervention jurisdictions and the prevalence-only communities. The number of total communities (n = 27) and the minimum of 150 children per community was selected as it allows detection of modest effect sizes of 0.35 or less, with a power of 80% and a critical level of 0.05 (two-sided) [27]. The number of children of 200 to be measured in the prevalence-only jurisdictions was selected to provide reasonable precision, with coefficient of variation (CV) of 14%-20% for prevalence estimates. The sample sizes for the intervention jurisdictions of 713 to 988 clustered within communities result in CV's of 3%–4%. In brief, child measurements included descriptive information (e.g., age, sex, race/ethnicity, and infant feeding history), anthropometry (height, weight, and waist circumference) [43], acanthosis nigricans screening [44], diet and activity (2-day Food and Activity Logs and 6-days of accelerometry) [45],[46], screen time [47], and sleep time [48]. Parent and household measures included domains such as income, education, household composition, and cultural affiliation. Measurement activities generally consisted of two visits. At the first visit, parents completed the questionnaires and were trained on the completion of the Food and Activity Log while the children were measured and fitted with an accelerometer. The second visit, approximately one week later, consisted of the retrieval of the Food and Activity Log and the accelerometer. Parents consented and children assented to participate. Participants received $20 for participation in most locations. Remuneration in Guam was increased to $40 and in Alaska to $50 based on jurisdictional preference. Institutional Review Board approval from the University of Hawai‘i, the University of Guam, the University of Alaska Fairbanks, and from the Republic of Palau was granted for the CHL study. Other jurisdictions ceded IRB authority to the University of Hawai‘i. Additional Institutional Review Board approval was not needed for this particular examination, as no personal health information was involved. For further details on the study design and other aspects of the study protocol, see Wilken et. al. [27].

### 2.2. Team

To conduct the prevalence survey, a local CHL measurement team was established in each of the eleven jurisdictions. With the exception of the FAS region, each of the jurisdictions had a lead site investigator. For the FAS, one lead site investigator was identified for all three countries due to limited resources and infrastructure. In addition to each lead site investigator, each jurisdiction had a coordinator [26]. Other staff was hired or contracts established with agencies with similar interests to implement the study recruitment and measurement activities.

### 2.3. Identification of Recruitment Processes, Strategies and Lessons Learned

In June 2015, after the completion of the last prevalence data collection, members of the CHL Program from across the region, which included the coordinators from each jurisdiction's local CHL measurement team, met in Honolulu, Hawai‘i for a week-long, face-to-face meeting. During this meeting, the team coordinators met for half a day to discuss the recruitment processes, strategies, and lessons learned. An initial list of recruitment strategies and lessons learned was generated for each jurisdiction at that meeting. Team coordinators were then instructed to return home to the jurisdiction to share the compiled strategies and lessons learned with other members of their measurement team to confirm agreement or modify.

### 2.4. Qualitative Data Analysis

To identify common recruitment strategies and lessons learned across the region, jurisdiction- specific strategies and lessons learned were reported by each jurisdiction representative and then combined by two of the co-authors, AY and MKF. Using a deductive approach, recruitment strategies were categorized as school-based if jurisdiction strategies mentioned terminology related to schools, school administrators, teachers, children (enrolled in school), or parents/caregivers (of children enrolled in school). All other recruitment strategies were labeled as community/agency-based. The recruitment strategies were then organized by the frequency reported by each jurisdiction. In addition, each jurisdictions summary of recruitment strategies mentioned specific methods and materials used. Any method or material mentioned by the jurisdictions were categorized and indicated. The lessons learned reported by each jurisdiction were clustered into lessons learned theme by similarity. For example, lessons learned related to being accommodating of parents schedule, flexible in scheduling events and helping with transportation as needed were categorized as “flexibility” due to their similarity. To maintain the uniqueness of each jurisdiction, data are presented by jurisdiction. The authors of this paper represent at least one member of each jurisdiction team and reviewed, organized, and confirmed the recruitment strategies and lesson learned themes.

## 3. Results

The study recruitment goals and number of children consented are presented in Table 2. Based on the number of children consented, the majority of jurisdictions met their minimum recruitment goal. Most jurisdictions averaged at least 20 children consented per measurement event.

**Table 2.** Number of Children (2 – 8 years of age) Targeted and Consented in the Children's Healthy Living Program Childhood Obesity Prevalence Survey.

Jurisdiction	Minimum Recruitment Goal (n)^a^	Number of Measurement Events (n)	Number of Consented (n)^b^	Mean Consented per Event (n)
Alaska	720	26	713	27
American Samoa	900	27	978	36
Chuuk	200	6	231	39
Commonwealth of the Northern Mariana Islands	900	44	924	21
Guam	750	34	885	26
Hawai‘i	900	51	988	19
Kosrae	200	7	207	30
Republic of Palau	200	8	214	27
Pohnpei	200	4	212	53
Republic of the Marshall Islands	200	8	218	27
Yap	200	7	205	29
Total	5,370	222	5,775	26^c^

a Recruitment goals varied by jurisdiction and the number of communities participating (Alaska = 4 communities, American Samoa = 6 communities, Commonwealth of the Northern Mariana Islands = 6 communities, Guam = 5 communities, and Hawai‘i = 6 communities). The desired number of participants was 180 per community with a minimum number of 150 per community in the intervention jurisdictions in order to assess change. For Alaska only, where the number of communities was limited to 4 due to resources, the desired number of participants was 200 per community with a minimum number of 180 per community. The minimum number in each jurisdiction of the Freely Associated States of Micronesia was 200 children in order to determine prevalence.

b Children were consented after screening for eligibility (e.g., age 2-8 years old, resident of targeted community) with parents.

c Mean Total Consented per Event

### 3.1. Recruitment Process and Strategy

Each jurisdiction composed their local CHL measurement team. For example, American Samoa, Guam and Alaska built their teams from Cooperative Extension programs and included staff from their Expanded Food and Nutrition Education or 4-H Programs. CNMI partnered with the nursing program at the Northern Marianas College. The teams in Hawai‘i and the FAS region, where there was considerable (expensive) inter-island travel, were a hybrid of a travelling core team and a local team from partner programs such as Head Start/Early Childhood Education Centers. Multi-lingual team members were included in jurisdictions where languages besides English were spoken, including FAS, American Samoa, CNMI, and Guam. The process and strategies used by CHL for recruitment are presented in Table 3.

To facilitate the process, every jurisdiction established a local advisory committee comprised of representatives from partner agencies including schools (Head Start, Early Childhood Education, Department of Education, etc.), government agencies (Department of Health/Public Health, Mayor's Office, etc.), and non-governmental agencies (Private Daycares, Community Organizations, Parent Associations, etc.) to facilitate the process. With the exception of FSM and RMI, most jurisdictions used a combination of school and community/agency-based recruitment process. Head Starts/Early Childhood Education Centers were targeted by CHL because they were in common across jurisdictions and served young children from underserved populations. However, elementary schools were also included in certain jurisdictions (American Samoa, Guam and FAS). Across all jurisdictions, the recruitment process involved meetings with school and community/agency officials. Gaining approval from schools and organizations to recruit in targeted areas often consisted of multiple approval levels (i.e., directors, principals, other administrators, and teachers in school districts). Establishing relationships with high-level officials allowed the team to gain access to communities.

CHL established partnerships with organizations that shared common goals and missions for the health of young children with CHL, including Head Start/Early Childhood Education (all jurisdictions), the Supplemental Program for Women, Infants, and Children (CNMI and Alaska), the Boys and Girls Club (Alaska), the Island Food Community of Pohnpei, FSM, and the Housing and Urban Renewal Authority (Guam). Religious organizations, such as the Catholic Diocese in Guam and CNMI and churches in American Samoa, were also recruitment venues. Organizations agreed to make announcements about the project to increase CHL's credibility and reach, and organizational staff often assisted in recruiting parents/families.

The types of recruitment materials used and the method of delivering them are listed in Table 4. Face-to-face meetings were the most common form of recruitment across the region. These meetings occurred with administration heads, organization leads, cultural leaders, community leaders, teachers and parents. The most common type of recruitment material, used in seven jurisdictions, was letters addressed to organizations or to parents. These letters were always sent prior to face-to-face meetings or e-mail and telephone calls. Flyers and/or letters were often given out by teachers as a trusted leader. Methods that involved technology such as radio, texts and emails were not frequently used. Since CHL needed to recruit children within specific communities, mass media methods such as radio were not a preferred approach unless targeting was possible. The only exception was in RMI for one of the remote outer atolls where citizen-band radio communication was the only reliable method available for reaching the small community.

All participants were compensated for their time. However, the mode of compensation differed by location; this was decided under advisement of the local advisory committees. Gift cards were used in Hawai‘i and Alaska. Guam and CNMI used vouchers to local businesses. In American Samoa and FAS, cash was the preferred mode of remuneration. Compensation was also provided in some jurisdictions in forms of gift cards (Hawai‘i and Alaska) or vouchers (Guam) to members of partner organizations, such as teachers or the Head Start site, for assisting with the recruiting process and logistics.

**Table 3.** Recruitment Strategy in the Children's Healthy Living (CHL) Program Childhood Obesity Prevalence Survey by Each Jurisdiction.

	Recruitment Strategy	Alaska	American Samoa	Chuuk	Commonwealth of the Northern Mariana Islands	Guam	Hawai‘i	Kosrae	Republic of Palau	Pohnpei	Republic of the Marshall Islands	Yap
**School-based^a^**	Meet with school officials (face-to-face)	X	X	X	X	X	X	X	X	X	X	X
	Establish MOU/Gain Approval	X	X	X	X	X	X	X		X	X	
	Work with community partners (potential resource to base operations from)	X							X	X	X	X
	Schedule date with administrators and teachers	X	X	X	X	X	X	X	X	X		
	Meeting with administrators and teachers	X	X	X-		X	X	X			X	
	Meeting with parents/guardians			X	X	X	X	X		X	X	X
	Recruitment materials translated		X	X				X		X	X	X
	School distribute recruitment materials	X	X		X	X	X	X	X			X
	Advertise through community outlets								X	X	X	
	Remind parents of measurement date	X			X		X				X	
	Encourage parents to bring other eligible children	X					X					X
	Email reminders to announce at school meetings				X							
	Follow cultural protocols ^d^		X	X				X		X	X	
**Community^b^/Agency-based^c^**	Establish local advisory committees	X	X	X	X	X	X	X	X	X	X	X
	Identify organization	X				X	X					X
	Contact director	X			X	X	X					X
	Establish MOU/Gain Approval	X	X		X	X	X					X
	Schedule meeting (face-to-face)	X	X									X
	Set date	X			X		X					
	Provide recruitment materials	X	X		X	X	X					
	Post recruitment flyers	X				X	X					
	Contact parents/guardians				X	X	X					
	Follow cultural protocols ^d^		X		X		X					X

MOU: Memorandum of Understanding

a School: Any institution at which instruction is given in a particular discipline; an institution for educating children. This included preschools and elementary schools.

b Community-based organization: Public or private nonprofit (including a church or religious entity) that is representative of a community or a significant segment of a community, and is engaged in meeting human, educational, environmental, or public safety community needs.

c Agency-based organization: a business or organization established to provide a particular service, typically one that involves organizing transactions between two other parties.

d Cultural protocols included bringing a gift to chief, sharing food at meetings, or starting meetings with a prayer.

**Table 4.** Recruitment Materials and Methods Used in the Children's Healthy Living Program Prevalence Survey Organized by Each Jurisdiction

	Methods				Materials			
Jurisdiction	Telephone	Radio	Door-to-door	Meetings	Newspaper	Email	Flyers	Letters
Alaska				X			X	X
American Samoa				X				X
Chuuk	X			X				X
Commonwealth of the Northern Mariana Islands	X			X		X	X	X
Guam	X		X	X			X	X
Hawai‘i	X			X		X	X	X
Kosrae				X				X
Republic of Palau		X		X	X			
Pohnpei	X		X	X				X
Republic of the Marshall Islands		X	X	X			X	
Yap				X			X	X

## 4. Discussion

The purpose of this paper was to document the recruitment processes, strategies, and lessons learned for conducting a multi-jurisdiction childhood obesity prevalence survey across the US Affiliated Pacific region. Recruitment is one component of the many layers of research. It requires multifaceted approaches that are tailored to the recruitment locale. This is especially apparent for CHL, which covers an expansive and diverse region. There is no single technique that was optimal, and each jurisdiction had to determine the approach that worked best for it. This highlights the region's diversity, unique sociocultural environment, and the inability for a one-size-fits-all approach to work.

One of the findings of this evaluation was the importance of ensuring alignment between upper management (administration) and those on the “ground” (e.g., in the classrooms) in partner organizations. If either individual did not view participation in the prevalence survey as a priority, it impeded participation. In general, school administrators were supportive of CHL's request to recruit in their schools, and teachers served as conduits for disseminating recruitment materials to parents/caregivers and children. Jurisdictions also found that it was important to hold orientation meetings either through Parent-Teacher Associations (CNMI) or Parent Councils (Guam) or independently (Chuuk, Hawai‘i, Kosrae, Yap) for parents not only to share about the program but to ensure that measurement events would be scheduled to accommodate busy schedules.

The preference to work with school-based organizations was reflective of the need to recruit young children within each community. Although there were instances when schools enrolled students from outside the CHL community of interest, generally schools were an effective location for recruitment. Community-based events required significantly more screening, since they were open to the public.

It was important to align measurement events with other organizational events, such as health fairs, to garner participation in the community. Through aligning with other organizations known in the community, CHL's credibility was increased, an important feature of community-based research [49], [50]. In the smaller jurisdictions, such as CNMI, Guam, and the FAS, the local CHL team had previously established relationships with leaders of organizations, which helped to facilitate measurement events.

Following cultural protocols in specific jurisdictions was also important for recruitment. Oral cultural protocol, reflective of the Pacific region [51], was demonstrated in the primacy of face-to-face meetings as a tool to disseminate recruitment materials and the low appeal/use of technology-driven approaches. Examples include opening meetings with prayer in Native Hawaiian communities and receiving a blessing from the village matai (chief) in Samoan communities. In Yap, it was important to bring a gift of coffee, pastry or betel nut (referred to as kaptalwa) for outer-island chiefs before asking permission to recruit. Food was generally expected and provided at events to demonstrate respect and appreciation.

Although focusing on individual recruitment in intervention programs, Hooks et al. reported that there are eleven components to a successful recruitment campaign [28]. These components are 1. Segment or target the audience; 2. Establish trust in the program and staff; 3. Promote an awareness of the project in the target audience; 4. Enhance the perceived and real benefits of participation; 5. Minimize the perceived and real costs of participation; 6. Enhance the capability and perceived self-efficacy of the participants; 7. Establish supportive bonds between previous and subsequent participants; 8. Ask for a decision and a commitment to participate; 9. Help participants solve the problems of initial participation; 10. As opportunities are presented, help the participants meet other needs to establish an exchange of responsibility; and 11. Work with the participants in solving problems until their participation ends [28]. With the exception of components 9 – 11, which apply to participants participating in an intervention program over time, each of the jurisdictions addressed the components in the recommended protocol of Hooks et al. [28]. Jurisdictions knew their target audiences (2 – 8 year olds), established or re-visited relationships with partner organizations serving young children, disseminated information, and met with organization representatives and participants (parents/caregivers) to share information and enhance perception of the program. CHL staff knew to be clear about expectations of participation (e.g., informed consent), prior to gaining participant agreement to participate. Each jurisdiction approached these components through customized strategies, which resulted in minimum recruitment goals being met for the survey.

This study was limited in not asking participants of the prevalence study about the effectiveness of various recruitment strategies. The results shared here are reflective of the views of the staff regarding the process. It may be that what the staff viewed as a useful method for recruitment was not similarly perceived so by participants.

### 4.1. Lessons Learned

The lessons learned for the CHL jurisdictions centered around four themes--community organization buy-in, communication and advance planning, local travel and site traits, and flexibility (Table 5).

Many jurisdictions reported that establishing relationships, partnerships, or gaining approvals from organizations with similar missions was important. This included establishing local advisory committees comprised of leaders from partner organizations.

Departments of Education, which administers Kindergarten–12th grade, were not major recruitment venues for Alaska, Guam and Hawai‘i due to procedural challenges, different organizational priorities, and insufficient time to find a common plan. This meant that CHL teams in those jurisdictions needed to partner with other federal programs such as Head Start, Supplemental Program for Women, Infants, and Young Children or local governmental organizations such as the Borough (Alaska) or Mayor's office (Guam) for measurement implementation. Being informed of organizational priorities early in the recruitment process was important to determine the best approach for working with organizations. For example, since CHL did not hold measurement events on Department of Education grounds, in Guam we were still able to provide information and invitations to students' parents about measurement events in the community.

Coordination and communication of expectations, including the accommodation of parents/caregivers and organization needs, were important to holding successful measurement events. Although developing plans for conducting measurement events were important, jurisdictions noted the importance of being flexible and open to adapting plans and schedules in order to increase the likelihood of attendance at events. This was especially apparent in some of the jurisdictions that required measurers to cover great distance via land (Alaska) or ocean (RMI, Yap and Hawai‘i). Success required an awareness of travel limitations (e.g., flights between or within some jurisdictions departed twice weekly, not daily), costs, and institutional and grant limitations on travel and recruitment procedures.

**Table 5.** Recruitment Lessons Learned in the Children's Healthy Living Program Childhood Obesity Prevalence Survey Organized by Themes and Jurisdiction

	Lessons Learned	Alaska	American Samoa	Chuuk	Commonwealth of Northern Mariana Islands	Guam	Hawai‘i	Kosrae	Republic of Palau	Pohnpei	Republic of Marshall Islands	Yap
Community Organization buy-in	Follow administrative hierarchy for obtaining organization approval	X	X			X	X					X
	Form relationship with partners (including local advisory committees)	X		X	X	X	X		X			X
	Face-to-Face meetings with leadership	X	X	X			X	X				
	Align goals to meet partners' goals						X		X	X		
	Work with trusted community partners	X			X		X			X	X	
	Remuneration for participation/assistance					X	X			X		
Communication and Advance Planning	Set clear expectations	X			X							
	Hand deliver recruitment materials		X		X	X	X	X				
	Follow-up with telephone calls				X		X					
	Communicate with school staff	X			X		X					
	Hold interest meetings							X				
	Train staff to be good communicators						X			X		
	Plan in advance for remote sites	X									X	
Local Travel and Site Traits	Set minimum number to hold an event as a means to control cost	X										
	Work with established community events	X					X					
	Know flight and boat schedule to remote locations	X					X				X	X
	Identify and adhere to cultural protocol							X				X
	Identify locations that would allow for the best turnout (church, school, health clinic, or other central location)	X					X			X	X	X
Flexibility	Continually get feedback from partners and parents			X			X					X
	Accommodate schedules, locations, and preferences of parents and partners	X		X	X	X	X		X	X		
	Be flexible about location and time	X				X	X					
	Prepare to recruit on day of measurement event						X				X	
	Help with transportation when needed										X	

## 5. Conclusion

This paper provides a reference and a springboard for others seeking to support or conduct public health research in resource-limited and geographically isolated locations. For remote locations, identifying an organization that provides an infrastructure for integrating measurement activities may be a sustainable and cost-effective manner in which to conduct monitoring of childhood obesity prevalence.
